# Optimizing a Personalized Health Approach for Virtually Treating High-Risk Caregivers of Children With Neurogenetic Conditions (Project WellCAST): Protocol for a Randomized Controlled Trial

**DOI:** 10.2196/64360

**Published:** 2025-06-25

**Authors:** Bridgette Kelleher, Kaleb Emerson, Lyndsey N Graham, Veronika Vozka, Anne Wheeler, William Fadel, Daniel Foti, Isha Metzger, Mandy Rispoli, Wendy Machalicek, Laurie McLay, Sean Lane, Wei Siong Neo, Allie Carter, Lisa Brown, Jennifer Brown, Laura Lee McIntyre, Elizabeth Salwitz, Gloria Dietz, Riley Naughton, Katlyn Peek, Nicole Hollins, Emma Woodford

**Affiliations:** 1 Purdue University West Lafayette, IN United States; 2 RTI Research Triangle Park, NC United States; 3 Indiana University Indianapolis, IN United States; 4 Georgia State University Atlanta, GA United States; 5 University of Virginia Charlottesville, VA United States; 6 University of Oregon Eugene, OR United States; 7 Faculty of Health University of Canterbury Ilam, Christchurch New Zealand; 8 University of Missouri Columbia, MO United States; 9 University of Canterbury Ilam, Christchurch New Zealand

**Keywords:** caregivers, neurogenetic conditions, telehealth, precision health, community-academic partnerships, ecological momentary assessment, mental health, motivational interviewing, clinical psychology, parent coaching

## Abstract

**Background:**

Even before the COVID-19 pandemic, caregivers of children with rare neurogenetic conditions (NGCs) experienced physical and mental health challenges. These challenges escalated during the COVID-19 pandemic due to crisis-level breakdowns in support services. Tele–mental health and parenting support services expanded rapidly in response to the COVID-19 pandemic and may be well suited to facilitate necessary support interventions for NGC caregivers. However, it remains unclear how to match these evidence-based interventions to individual NGC caregivers’ needs.

**Objective:**

Project WellCAST (Supporting Well-Being of Caregivers via Telehealth) is an early-phase clinical trial designed to prospectively test which evidence-based telehealth interventions best meet the needs of NGC caregivers.

**Methods:**

Interested and eligible NGC caregivers are enrolled in a 24-week program with 5 phases, including baseline (2 weeks), support program (12 weeks), and follow-up (2 weeks) periods; a 4-week gap separates the phases. Caregivers participate in 2 randomizations, namely support program assignment via a precision health algorithm versus quasi-random assignment and motivational coaching by another NGC caregiver and project staff member (“peer coaching”) versus standard check-ins by a staff member who is not an NGC caregiver (“staff coaching”). Virtual support programs include acceptance and commitment therapy, dialectical and behavioral therapy, culturally informed cognitive behavioral therapy, research units in behavioral intervention, naturalistic communication intervention, Durand sleep intervention, and self-guided resources. A subset of caregivers will participate as waitlist controls before engaging in support programs. We developed and optimized a personalized health decision tree algorithm that matches caregivers to telehealth support programs. We then proceeded to test the feasibility and efficacy of algorithm-assigned support programs across 4 waves of data collection, relative to quasi-random assignment and waitlist controls. During each wave, the personalized health algorithm relies on 2 weeks of baseline data collection using clinical tools and innovative smartphone-based ecological momentary assessments. Across waves, we also test the efficacy of a motivational peer-to-peer coaching protocol, deployed by trained NGC caregiver staff, in enhancing support program uptake and clinical outcomes.

**Results:**

Four waves of data collection are scheduled for August 2023 to September 2025. Preregistered analyses will contrast feasibility, efficacy, and acceptability across algorithms and coaching assignments. Multiple waves of data collection will allow us to continually optimize the algorithm and test incremental improvements across project phases. Secondary analyses will probe the feasibility and efficacy of individual evidence-based support programs and peer coaching.

**Conclusions:**

Project WellCAST will test whether a digital personalized health decision tree algorithm and peer coaching protocol can prospectively enhance telehealth support program outcomes among NGC caregivers. This project is relevant to the specific population of NGC caregivers and may also inform how brief digital assessments, precision health tools, and community-academic partnerships can enhance the public health response to mental health crises across other high-need populations.

**Trial Registration:**

ClinicalTrials.gov NCT05999448; https://clinicaltrials.gov/study/NCT05999448 and OSF Registries 10.17605/OSF.IO/8WNDP; https://osf.io/8wndp

**International Registered Report Identifier (IRRID):**

DERR1-10.2196/64360

## Introduction

### Overview

Although tele–mental health and parent coaching services are increasingly available, it remains unclear which types of support programs are best suited to various populations and how to best deliver these support programs. One population particularly well suited to telehealth support programs is caregivers of children with neurogenetic conditions (NGCs). Here, we defined NGCs as genetic conditions that affect cognitive, psychosocial, and physical development; examples include named conditions, such as Angelman syndrome, Prader-Willi syndrome, and Williams syndrome, as well as rare and ultrarare conditions with lower prevalence rates. These NGC caregivers are vulnerable to elevated stress and mental health challenges due to often extreme caregiving burden; for example, they may experience geographic isolation from specialty support clinics, exhibit elevated levels of parenting stress, and manage complex child behavioral and medical needs with limited structural resources [1-3]. Project WellCAST (Supporting Well-Being of Caregivers via Telehealth) is a National Institutes of Health–funded clinical trial that aims to prospectively develop and test the efficacy of a precision health decision tree algorithm for routing NGC caregivers to evidence-based telehealth supports, based on their unique clinical and lifestyle profiles. Project WellCAST also tests the efficacy of community-engaged peer-to-peer coaching protocol in enhancing the feasibility and efficacy of digital supports. Here, we provide the rationale and protocol for Project WellCAST, including anticipated outcomes and impact within and beyond NGC caregiving communities.

### Project Background

Although telehealth support programs have existed for some time, telehealth services expanded rapidly in response to the COVID-19 pandemic [4], providing new opportunities for increased access to mental health programs [5,6]. One population for whom telehealth services offer critical benefits are NGC caregivers. Although the elevated mental health needs of NGC caregivers predated the COVID-19 pandemic [7], the pandemic caused them to face crisis-level breakdowns in support and intervention services that have continued to impact families [8]. These COVID-19 pandemic–related impacts were, in part, driven by social isolation, financial strain, and reductions in face-to-face support programs, which compounded an already fragmented and challenging landscape of care [2,3]. The COVID-19 pandemic–related interruptions required caregivers to take on even more responsibility as they managed urgent educational, vocational, and functional needs of their children [3]. The negative impact on caregivers’ health and well-being continues to this day [8]. However, despite their elevated need, caregivers rarely receive support for their own mental health. Any psychological support they do receive is typically disconnected from their child’s care plan and delivered by practitioners with little-to-no expertise in the needs of NGC families [9,10]. Consequently, accessible and scalable digital interventions are needed to support the intersectional needs of NGC caregivers and prevent an accelerated breakdown in their mental health, particularly in the wake of the COVID-19 pandemic.

In part due to the rapid and dramatic uptake in telehealth methods during the COVID-19 pandemic, many questions remain regarding the circumstances and populations for which telehealth services are most effective [6], including but not limited to services for NGC caregivers. Although a variety of evidence-based telehealth support programs are available, it is not always clear which programs should be provided to which patients. This issue has long motivated the psychiatry and psychology literature, though many randomized controlled trials continue to examine broad treatment effectiveness [11,12] rather than investigating treatment personalization protocols [13]. Precision health literature guides our efforts to move toward support program personalization for NCG caregivers, with promising examples of prognostic models in other populations [14].

One challenge to the effective prospective assignment of patients to treatments is the issue of measurement. Often, mental health providers base selection on limited intake information and through subjective assessment guided by broad symptom checklists or patient-reported symptoms. As an expansion of this approach, precision mental health care aims to optimally match patients to existing evidence-based treatments through the use of demographic, clinical, and dispositional information [14]. Tools such as daily diaries and ecological momentary assessments (EMAs) hold promise for providing temporally dense information about participants’ experiences, beyond the contributions of clinical assessments alone. EMAs are brief questionnaires aimed at gathering real-time data on the participants’ experiences that reduce the challenges of retrospection [15]. Using EMA data can provide insights into fluctuating daily experiences and momentary affects, as well as information about practical day-to-day constraints within their lives that may affect support program uptake (eg, caregiving burden, health behaviors, and social support). However, to date, tools such as EMA have not been prospectively used to match patients to support programs based on their personal health profiles.

Another consideration for the effective assignment of support programs is the impact of sociocultural and practical issues on a patient’s willingness and ability to engage in tele–mental health treatment. For example, several studies have documented that telehealth does not universally improve accessibility and may, in some cases, enhance preexisting disparities by disrupting trust and rapport, distancing communities with poor technology literacy or access, and introducing concerns about data security and privacy [16]. These health care gaps are amplified among Black and other minority families who are less likely to receive mental health care due to discrimination, racism, and historic injustices against Black people in health care settings [17].

One potential solution to the disrupted rapport in telehealth-based programs is to implement community-centered and motivational support programs, such as participation enhancement interventions aimed at addressing barriers to support program participation and enhance motivation and adherence to treatments [18,19]. These support programs may be particularly beneficial for caregivers of children with NGCs due to isolation and disconnection from social support that may accompany their child’s complex needs [9,10]. Peer support is known to provide benefits to caregivers [20,21], though to be most useful, perceived need and received support must align [22]. There is also some evidence that peer support may have differential impacts on certain subgroups of parent caregivers (eg, individuals with higher baseline anxiety [23]), though more evidence is needed to determine which participants might benefit most from peer support. Thus, peer support interventions may positively impact willingness and engagement in tele–mental health programs for some caregivers while simultaneously being an extra unneeded hassle for others. Although these programs have demonstrated broad efficacy across several neurodevelopmental populations, they have not been explicitly tested in substantial NGC caregiving samples. Given that coaching programs require extra time and resource commitments from both families and professionals, and many participants effectively engage in intervention without peer support, it is important to determine which participants would benefit from this extra layer of support.

### This Study

Project WellCAST (NCT05999448) is an early-phase clinical trial designed to prospectively test which evidence-based telehealth interventions best meet the unique needs of NGC caregivers. We accomplish two specific aims: (1) develop and optimize a personalized health decision tree algorithm to match NGC caregivers to digital health programs using a combination of standard clinical tools and smartphone-based EMA data, and (2) test the efficacy of peer-to-peer coaching, deployed by trained NGC caregivers using an evidence-based motivational interviewing protocol, in enhancing support program uptake and clinical outcomes. Here, we describe the protocol methods and anticipated impact of this project, both for NGC caregivers and the broader field of precision mental health.

## Methods

### Study Design

Project WellCAST uses a randomized control design with waitlist control to test the effectiveness of both (1) a precision health algorithm and (2) a participation enhancement intervention on improving clinical outcomes for NGC caregivers. It is anticipated that between 500 and 750 caregivers will enroll in WellCAST across waves, with a minimum enrollment of 500 participants, including 50 waitlist controls, to adequately power primary analyses. SPIRIT (Standard Protocol Items: Recommendations for Interventional Trials) reporting guidelines were used in the preparation of the Project WellCAST protocol [24].

Project WellCAST is deployed across 4 waves. During waves 1 and 2, participants are randomly assigned to be routed to support programs using either a preregistered precision health algorithm (algorithm 1) or quasi-random assignment. During waves 3 and 4, participants are assigned to support programs using optimized algorithms (algorithms 2 and 3, respectively). Across waves 1 to 4, participants are randomized to either a participation enhancement intervention delivered by an NGC caregiver coach (peer coaching) or similarly timed check-ins by non-NGC caregiver staff (staff coaching).

At the time of the initial protocol submission, Project WellCAST has completed waves 1 and 2 (fall 2023 and spring 2024); waves 3 and 4 will be conducted in fall 2024 and summer 2025, respectively. Thus, across waves, 25% of participants are assigned to each algorithm category (quasi-random, algorithm 1, algorithm 2, and algorithm 3), and 50% of participants are assigned to each peer coaching category (peer coaching and staff coaching).

### Ethical Considerations

#### Overview

Project WellCAST was determined to present no more than a minimal risk of harm to participants. The study was approved by the Purdue University Institutional Review Board (IRB) on March 3, 2023 (2022-1058); collaborating sites deferred oversight to Purdue as the single IRB of record. Participants provide informed consent before engaging in baseline data collection and later provide informed consent specifically for their assigned support program before engaging in sessions.

#### Compensation

Caregivers are paid US $100 for completing assessments. There are 5 sets of forms total, and the amount of compensation increases over time to maximize response (baseline: US $10; start of programs and mid support programs: US $15 each; end of programs: US $30; and follow-up: US $30). Participants are not compensated for time spent in support programs. Participants who are participating in the waitlist control receive compensation as they would if they participated in a support program. Payment is delivered using Amazon gift cards after participants complete each set of forms, and support programs are delivered free of charge.

#### Privacy and Confidentiality

Participants provide a variety of personal health information data, including medical and genetic records and psychiatric diagnostic information. We are not subject to the Health Insurance Portability and Accountability Act (HIPAA) guidelines, as we are not a medical entity. However, we use HIPAA-compliant data storage tools and procedures whenever possible. Data are stored on password-protected and encrypted devices, HIPAA-compliant Microsoft Box folders, and the HIPAA-compliant data management system REDCap (Research Electronic Data Capture; Vanderbilt University) [25,26]. Participant data are labeled by identification numbers rather than name, wherever possible. Only key study personnel have access to REDCap, where identification numbers are stored alongside participant information. Otherwise, identification numbers are stored separately from personal information.

#### Plan for Documenting Potential Protocol Modifications

Potential deviations from protocol and protocol modifications are logged into a protocol deviation log by the research team. Applicable protocol modifications will be submitted to the IRB for review at each instance. The research team will complete a yearly audit of the registered protocol on all platforms.

### Trial Registration

Project WellCAST was registered on ClinicalTrials.gov on August 21, 2023, and the first algorithm was registered on Open Science Framework (OSF) Registries on August 30, 2023, before running the first algorithm assignment. The first participant consented to a support program on August 31, 2023, and the first participant started the support program on September 11, 2023. Supplemental analytic plans were preregistered on OSF Registries on October 18, 2023, to further detail the ClinicalTrials.gov registration. Wave 1 participants consented to baseline data collection starting June 28, 2023, and started baseline data collection on August 7, 2023. Baseline data were collected before any participant consented to support programs and were not analyzed or used to route participants until after registration was complete.

### Recruitment

Caregivers of children with NGCs are recruited through various sources to maximize the potential participant pool. Recruitment strategies include social media advertisements, sharing materials with genetic clinics and providers, mailers by patient advocacy groups, emails to condition-specific email distribution lists, and recruitment through registries. Inclusion criteria are (1) legal guardian of an individual aged 2 to 35 years with a documented NGC associated with intellectual or developmental disability that is not primarily characterized by neurodegeneration, (2) fluent in English, (3) interacts with the individual with an NGC most of the time, and (4) resides in the United States. Participants are excluded if they either (1) express severe psychiatric illness not indicated for the interventions offered (eg, suicidal, psychotic thoughts, mania, or significant substance use) or (2) are currently in active therapy or receiving support that would conflict with, or be redundant with, the interventions offered.

### Screening

Interested participants may scan a QR code on recruitment materials or receive a link via email to a digital prescreening survey via REDCap [25,26]. Once complete, research staff members who are trained in clinical assessments contact interested caregivers by phone to conduct a screening interview and to determine eligibility. The research staff member verifies demographic information and assesses the participant’s recent mental health history using the Mini-International Neuropsychiatric Interview [27]. If the participant is determined eligible to participate, the research staff member provides instructions on how to sign electronic consent forms for the baseline phase of the project in REDCap and begins the baseline phase of the study. Participants separately provide consent to their specific support program after being assigned a program arm during the routing phase.

### Decision Tree Algorithm Development

#### Overview

The decision tree algorithm was designed to function as a clinical decision tree. The rules and explanations for the algorithm are coded into an R program, and each output is reviewed by our biostatistics team to ensure that the algorithm is working correctly.

#### Initial Decision Tree Algorithm

The algorithm, decision nodes, and thresholds are all preregistered on OSF Registries before use to promote transparency, explainability, and interpretability. Support program assignments are based on clinical and lifestyle data provided by participants in the 2-week baseline period. For example, in algorithm 1, participants who report high levels of emotional dysregulation, depression, stress, or anxiety will be eligible for adult-focused mental health programs. Once eligible for adult-focused mental health programs, participants are assigned to either individually delivered acceptance and commitment therapy (ACT) or group-based dialectical and behavioral therapy (DBT) based on their initial data. Participants with higher emotional dysregulation scores are routed to DBT, whereas participants with below-threshold levels of emotional dysregulation are routed to ACT. This threshold was determined via multiple data sources. A primary aim of the study is to evaluate and optimize the efficacy of these algorithms, and we anticipate that support program assignment criteria will evolve following preplanned algorithm optimizations, as will be detailed in peer-reviewed manuscripts describing study results. The full descriptions of all decision nodes and thresholds for each iteration of the algorithm are available in algorithm preregistrations on OSF Registries. We also anticipate that further justification for the support program assignment will be discussed in the peer-reviewed manuscripts describing study results after the trial has concluded.

#### Minimizing Bias

Any algorithm or assessment tool will exhibit bias toward its normative sample. To minimize this, we prospectively implemented several protective measures that are also aligned with guiding principles for addressing algorithm bias [28]. We planned algorithm optimization via a CASCADE (community-engaged approach for scientific collaborations and decisions) panel at 2 preplanned intervals. The CASCADE panel process was intentionally designed to minimize biases. The key pillars of the CASCADE approach include centering community input, minimizing opportunities for cognitive biases in decision-making, and centering diverse experiences and perspectives, particularly of individual patients [29]. CASCADE panels include representation from individuals with lived experience in the trial’s target population (eg, rare disorder caregivers) present and engaged in the process. A specific example approach for addressing bias during CASCADE panels includes examining scores and data trends for minoritized populations within our sample to minimize the potential for exacerbated disparities. We anticipate that future applications of our final algorithm will require substantial interrogation for generalizability to any new participant group.

### Routing Procedures

Participants are matched to a combination of digital health support programs using a multistep process indicated by the data provided in the baseline phase. First, research staff not involved in participant support programs generate an eligibility panel for each participant that contains feasible and appropriate programs based on broad rule out criteria. For example, participants who do not identify with minority groups will not be eligible for programs focused on race-related trauma. Participants with children aged >8 years are not eligible for certain child-focused programs designed for young children. The preregistered algorithm is then used to generate a sequential list of participants’ recommended programs, based on their baseline data.

During waves 1 and 2, the first randomization is to algorithm 1 or quasi-random assignment. Participants randomized to algorithm 1 are assigned their algorithm-indicated support program. If practical issues prevent them from engaging with their top program (eg, lack of availability during group times), they are offered the next indicated support program. Participants randomized to quasi-random assignment are assigned to a support program using a random number sequence generated by a member of the biostatistics team who is not involved in participant management or intervention deployment. Similarly, if practical constraints prevent their first program from being feasible, they are moved to their next program indicated by the random number sequence. Whether the participant received their top assignment is recorded and integrated into statistical analyses. Finally, all participants are randomized to either peer coaching or staff coaching protocols using a random number generator. During waves 3 and 4, participants continue to be randomized to peer coaching or staff coaching. All participants receive algorithm 2 during wave 3 and algorithm 3 during wave 4. Care providers are masked to the status of peer coaching and algorithm assignment. Peer coaches are masked to the status of algorithm assignment. Data managers and statisticians evaluating study outcomes are provided deidentified data and are not involved in support program–related study operations. Research team members evaluating study outcomes are also masked to assignments.

A subset of participants is also assigned to a waitlist control to allow for the evaluation of treatment efficacy and quantify potential attenuation of responses with repeated assessment [30]. Participants assigned to waitlist controls contribute identical data to active participants and receive the same remuneration. They are routed to support programs or resources during the next wave.

### Support Programs

#### Support Program Categories

##### Overview

Support programs fall into one of four categories: (1) live telehealth intervention focused on caregiver well-being; (2) live telehealth intervention focused on a child’s social communication, behavioral, or sleep challenges; (3) self-guided resources; or (4) waitlist control.

##### Live Telehealth Intervention Focused on Caregiver Well-Being

Participants assigned to receive a live telehealth intervention focused on caregiver well-being are assigned to one of the interventions described in the subsequent sections.

###### ACT Intervention

Participants who are routed to ACT [31] receive an adapted 12-week version of the treatment containing the 6 major components of ACT. Each session is 60 minutes long and delivered via Zoom (Zoom Communications, Inc). ACT is administered individually between a supervised psychology doctoral student and a participant. In each session, clinicians review new skills and practices in line with the participant’s stated goals and review their practice of the skills in their daily life. The sessions are highly structured, while allowing clinicians flexibility with the examples and specifics of session content, so that the content can be most relevant to the participant [31].

###### DBT Skills Training

Participants assigned to DBT [32,33] receive an adapted 12-week version of the treatment. DBT is administered as a group therapy, with up to 2 doctoral students and 12 participants who meet once weekly for 90 minutes for 12 weeks. The skills sessions, based on the 4 core modules of DBT, are conducted in a condensed format to suit the 12-week format of DBT. The skills are curated and tailored to the needs of caregivers of children with NGCs. Some skills from the typical set are omitted to fit in with the abbreviated timeline [32,33].

###### Culturally Informed Cognitive Behavioral Therapy

Participants who identify as Black are eligible to be routed to an individual program or an additional, group version of culturally informed cognitive behavioral therapy [34,35]. This is an evidence-based support program for families affected by traumatic life events and includes psychoeducation, active coping skills, narrative processing, and exposure to traumatic memories [34,35]. The individualized form of this program is administered in once weekly 60-minute-long sessions, over 12 weeks, and delivered via Zoom. The group form is administered in a once weekly 90-minute-long format, with up to 10 participants. The group form is offered to any Black participant, in addition to another support program they were routed to (eg, participants could be in both ACT and this group).

##### Live Telehealth Intervention Focused on a Child’s Social Communication, Behavioral, or Sleep Challenges

Participants assigned to receive a live telehealth intervention focused on parenting techniques are assigned one of the interventions described in the subsequent sections.

###### Research Units in Behavioral Intervention

Research units in behavioral intervention (RUBI) [36] parent education is an empirically supported intervention that may help prevent and decrease the likelihood of worsening challenging behaviors for children [36]. This manualized program was adapted to a 12-week group-based program and teaches caregivers strategies for preventing and addressing their child’s mild to moderate challenging behavior, such as aggression and tantrums, while teaching appropriate caregiver behavior, such as communication and following a daily schedule with support. Each weekly session is 90 minutes long, consists of up to 4 participants and 2 group leaders, and is delivered via Zoom. Participating parents follow a workbook and are asked to complete weekly homework, which is reviewed at the next week’s session.

###### Naturalistic Communication Intervention

Participants assigned to the naturalistic communication intervention [37] receive an empirically supported intervention that helps promote their child’s early communication behaviors [37]. It is adapted into a 12-week program and is a 60-minute-long session, conducted individually between an interventionist and the participant. Each session aims to build on the child’s interests and preferences and promote communication behavior. This virtual session takes place during daily home and play routines.

###### Durand Sleep Intervention

Participants assigned to the Durand sleep intervention [38,39] engage in an evidence-based sleep protocol that provides educational content in a 12-week program for telehealth use. This intervention may help prevent or decrease sleep problems for the child. This is an every-other-week group program that offers caregivers resources and skills for identifying, preventing, and addressing their child’s sleep problems. On alternate weeks, the clinician checks in individually with the participant to support individual sleep challenges that the participant is facing [38,39]. The group sessions are 2 hours long with up to 7 caregivers, and the individual check-ins are 15 minutes long, alternating across the 12-week program.

##### Self-Guided Resources

Participants assigned to receive self-guided resources are provided with “The Project WellCAST Journal,” which was designed to be an active control condition to the live support program arms. This is a weekly reflections and resource guide for caregivers of children with NGC. During the 12-week guided practice, participants read about basic skills for well-being and mental health. Topics were selected to align with broad concepts that are addressed in many clinical psychology interventions, such as mindfulness, reflective listening, gratitude, breathing exercises, and motivating healthy living. Rare disorder caregivers working as project staff provided input on selected topics, specifically by helping narrow the list of options and suggesting formatting elements that would promote usability. Caregivers also added quotes about how topics relate to their lives as rare disorder caregivers. Each week’s entry includes space to reflect on past and current weekly topics, including practical exercises to bring topics to life. Participants do not submit the journal at the end of the study to maintain their privacy; however, they are asked to submit a log of when and for how long they used the journal.

##### Waitlist Control

Waitlist control participants complete assessment forms and are prompted to provide EMA data, without the presence of a support program. In the next wave, these participants are guaranteed enrollment in a support program.

#### Support Program Deployment

##### Overview

Support programs are deployed by trained graduate students and postdoctoral clinicians who are supervised by licensed professionals. Clinicians vary in previous exposure to NGC populations; however, all participate in preprogram, community-led training on NGC caregivers’ lived experiences, cofacilitated by NGC caregivers, to learn about community needs.

Participants are also randomly assigned to 1 of the 2 coaching programs as detailed in subsequent sections.

##### Peer Coaching

Peer coaches, who are NGC caregivers employed by the project, facilitate an evidence-based program for optimizing intervention engagement, grounded in motivational interviewing [18,19]. Sessions with a peer coach take 10 to 30 minutes and involve developing a collaborative plan via a “change plan” worksheet. This plan assesses participant-identified goals, identifies paths toward these goals, and proactively addresses barriers to support program attendance, persistence, and home practice. Peer coaches answer questions, troubleshoot barriers, and support accessing follow-up resources, such as connections with patient support organizations. Peer coaches were intentionally recruited to represent a range of NGC and demographic characteristics to align with our anticipated sample. Sessions are deployed via Zoom.

##### Staff Coaching

A research staff member, who is not an NGC caregiver and has not received training in participant enhancement interventions, conducts brief telephone check-ins at the same intervals as the peer coach to support participants by answering questions about the study or their needs for follow-up resources. Each staff coaching call is designed to take less than 5 minutes.

#### Study Procedures

**Figure 1:**
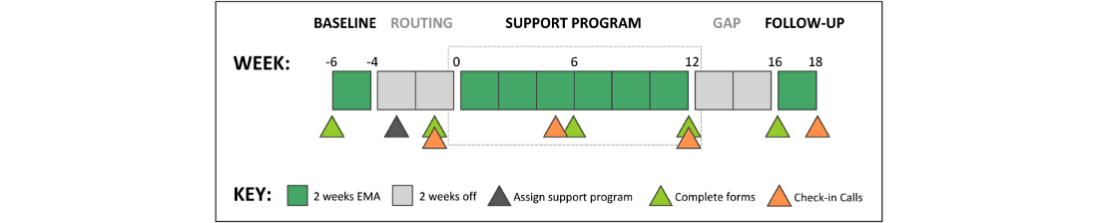
Study timeline. EMA: ecological momentary assessment.

Figure 1 depicts the 5 phases of each wave: baseline, routing, support program, gap, and follow-up. Participants only actively participate during the baseline, support program, and follow-up phases, with a 4-week gap during the routing and gap phases.

During the initial 2-week baseline phase, participants complete the first set of clinical assessment questionnaires and EMA surveys. They also complete their preprogram forms and first check-in call. Participants then engage in 12 weeks of their assigned support programs. Throughout their support programs, participants continue completing EMA reports, check-in calls with their coach (peer or staff), and clinical assessment questionnaires, at the timepoints indicated in Figure 1. Support programs are followed by a 4-week gap in study activities. Then, in the final 2 weeks*,* participants complete their last set of clinical assessment forms and 2 weeks of EMA reports. Participants also complete their last check-in call.

#### Data Collection

All data collection is deployed and collected using REDCap, a secure, web-based software platform designed to support data capture for research studies, providing an intuitive interface for validated data capture, audit trails for tracking data manipulation and export procedures, automated export procedures for seamless data downloads to common statistical packages, and procedures for data integration and interoperability with external sources [25,26]. Participants complete web-based data collection via personalized links sent via both text and email.

#### Measures

**Figure 2:**
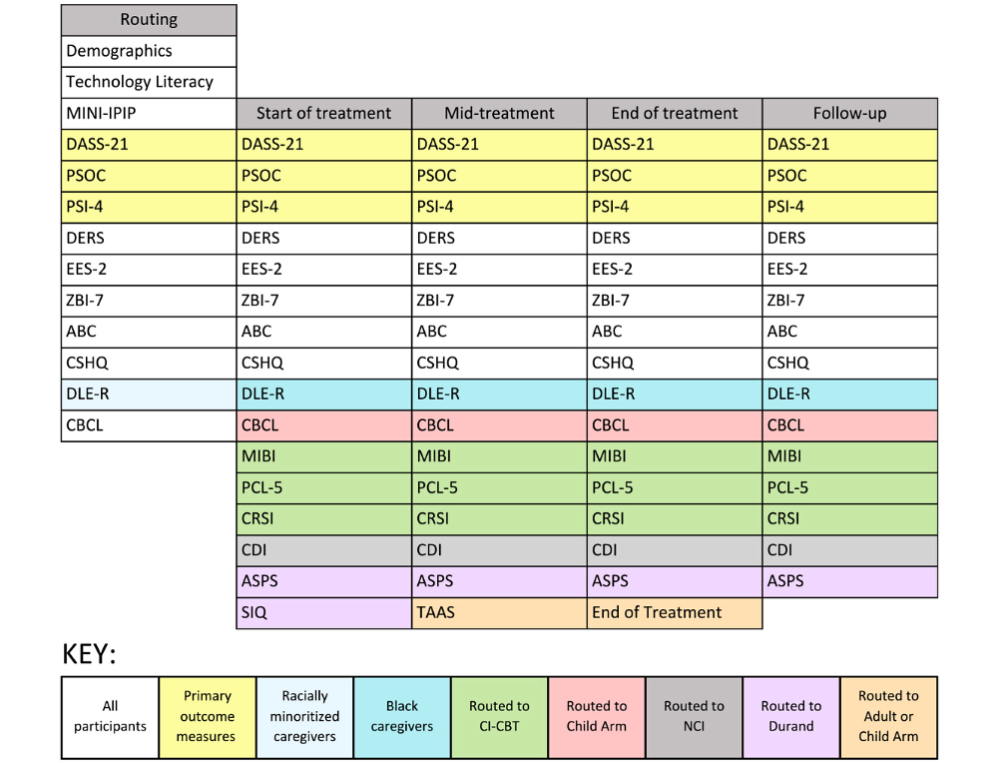
Primary and secondary outcomes. ABC: Aberrant Behavior Checklist; ASPS: Albany Sleep Problems Scale; CBCL: Child Behavior Checklist; CDI: Communicative Development Inventory; CI-CBT: culturally informed cognitive behavioral therapy; CRSI: Comprehensive Race Socialization Inventory; CSHQ: Child Sleep Habits Questionnaire; DASS-21: Depression, Anxiety, and Stress Scales-21 Items; DERS: Difficulties in Emotion Regulation Scale; DLE-R: Daily Life Experiences-Racism; EES-2: Emotion Efficacy Scale-2; IPIP: International Personality Item Pool; MIBI: Multidimensional Inventory of Black Identity; MINI: Mini-International Neuropsychiatric Interview; NCI: Naturalistic Communication Intervention; PCL-5: Posttraumatic Stress Disorder Checklist for Diagnostic and Statistical Manual of Mental Disorders, fifth edition; PSI-4: Parenting Stress Index, fourth edition; PSOC: Parenting Sense of Competency Scale; SIQ: Sleep Intervention Questionnaire; TAAS: Treatment Acceptability/Adherence Scale; ZBI-7: Zarit Burden Interview, 7-item short form.

Information for the questionnaires assessing primary outcomes of interest are presented in detail subsequently. A complete list of measures is presented in Table 1 and Figure 2.

**Table 1:** Citations for primary and secondary outcome measures.

Type	Measure	Abbreviation	Study
Primary	Depression, Anxiety, and Stress Scale-21 Items	DASS-21	Lovibond and Lovibond [40]
Primary	Parenting Stress Index, Short Form	PSI-SF	Abidin [41]
Primary	Parenting Sense of Competence Scale	PSOC	Ohan et al [42]
Secondary	Difficulties in Emotion Regulation Scale	DERS	Gratz and Roemer [43]
Secondary	Emotion Efficacy Scale-2	EES-2	McKay and West [44]
Secondary	Zarit Burden Interview, 7-item short form	ZBI-7	Higginson et al [45]
Secondary	Aberrant Behavior Checklist	ABC	Aman et al [46]
Secondary	Child Sleep Habits Questionnaire	CSHQ	Owens et al [47]
Secondary	Daily Life Experiences-Racism	DLE-R	Harrell [48]
Secondary	Multidimensional Inventory of Black Identity	MIBI	Sellers et al [49]
Secondary	Posttraumatic Stress Disorder Checklist for DSM-5	PCL-5	Blevins et al [50]
Secondary	Comprehensive Race Socialization Inventory	CRSI	Lesane-Brown et al [51]
Secondary	Child Behavior Checklist	CBCL	Mazefsky et al [52]
Secondary	MacArthur Communicative Development Inventory	CDI	Dale [53]
Secondary	Albany Sleep Problems Scale	ASPS	Durand [54]
Secondary	Sleep Intervention Questionnaire	SIQ	Durand [55]

#### Clinical Assessment Forms: Patient Report

Figure 2 describes clinical measures and deployment schedules, which vary depending on the phase the participant is in, the intervention assigned, and the participant’s demographic information. Primary outcomes of patient-reported clinical assessments are detailed in Textbox 1.

**Primary outcomes of patient-reported clinical assessments.**

**Depression, Anxiety, and Stress Scale-21 Items (DASS-21)**

DASS-21 [40] is used to assess psychological symptoms. It evaluates the severity of symptoms of depression (DASS-D), anxiety (DASS-A), and psychological distress (DASS-S) over the past week. Participants respond on a 4-point Likert scale from 0 (did not apply to me at all) to 3 (applied to me very much, or most of the time). In a sample of parents of children with autism, the total scale as well as all 3 subscales were found to have strong internal consistency (DASS-Total: α=.94, DASS-D: α=.88, DASS-A: α=.83, DASS-S: α=.92) [56].

**Parenting Sense of Competency Scale (PSOC)**

PSOC [42] is a 17-item scale that is used to measure caregiver self-efficacy. The PSOC has 2 subscales to measure a parent’s satisfaction and efficacy, which can also be combined to yield a total competence score. Example items include, “A difficult problem in being a parent is not knowing whether you’re doing a good job or a bad one.” Participants respond on a 6-point Likert scale from 1 (strongly disagree) to 6 (strongly agree). In a sample of parents of children with autism, the PSOC was found to have sufficient internal consistency (α=.79) [57].

**Parenting Stress Index-Short Form (PSI-SF)**

PSI-SF [58] is a 36-item self-report questionnaire used to assess perceived stress experienced by parents. Subscales include parental distress, parent-child dysfunctional interaction, and difficult child. Participants respond from 1 (strongly agree) to 5 (strongly disagree). The PSI-SF is known to be valid and reliable [41] and was later found to have strong internal consistency in a sample of parents of children with autism (α=.93) [56].

#### EMA: Patient Report

EMA surveys are deployed across baseline, support program, and follow-up periods. Participants receive links via text message for 3 EMA surveys per day; these links are sent randomly between 7 AM and 8 AM, 11 AM and 2 PM, and 7 PM and 8 PM, based on the participant’s local time. EMA surveys contain measures of sleep, daily activities, social interactions, caregiving responsibilities, stress, and affect. Each participant may contribute up to 336 EMA data points across the study period. Primary outcomes are described in Textbox 2.

**Primary outcomes of ecological momentary assessment (EMA) surveys.**

**Stress**

It is measured by asking participants “How STRESSED are you feeling right now?” Participants rate their stress on a visual analog scale of 0 (not at all) to 100 (extremely).

**The Positive and Negative Affect Schedule-Short Form (PANAS-SF)**

PANAS-SF [59] is used to measure positive and negative affect. Positive affect is measured by words such as “alert” and negative affect is measured by words such as “upset.” Participants are asked to indicate how much they feel each emotion right now, ranging from 1 (never) to 5 (always). Subscales of positive and negative affect have been validated in previous EMA studies (α=.90 and α=.89, respectively) [60].

#### Clinical Assessment Forms: Informant Reports

Clinicians and peer coaches provide ratings after each session, which summarize attendance, homework completion, and clinical symptoms. Clinicians and peer coaches also provide information on their perception of the participant’s support program acceptability at the end of the program. Primary outcomes are described in Textbox 3.

**Primary outcomes of informant-reported clinical assessments.**

**The Clinical Global Impression Scale (CGI)**

It is used to measure clinical impressions of severity (CGI-S) [61] and impressions of overall improvement since the start of support programs (CGI-I). Clinicians respond to a single item for each subscale, ranging from 1 (not at all ill or very much improved) to 7 (among the most extremely ill patients or very much worse since the initiation of treatment). The scale had strong internal consistency when used in a sample of adults with and without psychiatric conditions (CGI-S: α=.99; CGI-I: α=.94) [62].

**Support program mismatch**

It is assessed by asking clinicians and peer coaches to rate the fit from 1 (poor) to 5 (excellent).

**Homework completion**

It is assessed by clinicians during the sessions by asking participants if they have completed their homework. Following the session, clinicians report the participant’s homework completion rate.

**Dropout rates**

They are assessed by recording information relevant to participation and can include both passive and active dropouts. “Dropout” is defined as the discontinuation of support programs after consenting to and attending at least 1 session, including active discontinuation and patients lost to follow-up. Both active and passive dropouts are accounted for in data collection, with active dropouts referring to participants who actively express their preference to discontinue participation and passive dropouts referring to participants who passively discontinue engaging in support programs or data collection. Participants who are at risk for passively dropping out are emailed or texted by the study team up to 3 times with reminders and information regarding how to proceed with participation. If a participant wants to drop out, they can alert the clinician during a session or email the study team. The study team then follows up with the participant about their experience and how to alter the study’s fit for them. If the participant’s final decision is to unenroll, the study team will mark the date and the reason for the participant dropping out.

#### Data Quality

Researchers review critical data at least biweekly to ensure data quality. When student or staff clinicians are involved in the collection of sensitive data, licensed supervisors will review student or staff data. The biostatistics team runs regular data quality checks to ensure the completeness and correctness of all collected data. Any issues are addressed collaboratively by the biostatistics team and research team.

#### Patient Monitoring

All study team members can report patient concerns at any time using a centralized form that is immediately reviewed by project leaders. Supervisors are in at least weekly contact with clinicians to ensure ample access for reporting issues of concern; clinicians are also trained to reach out immediately if concerns arise. Clinical meetings with the investigator team and project intervention staff are held monthly, or as needed, to discuss any emerging concerns. Local supervisors meet at least weekly with all trainees involved in active intervention. All intervention leads are licensed mental health professionals and are prepared to make appropriate referrals to community mental health providers, crisis intervention, or safety authorities, as needed. During meetings, clinical staff and supervisors discuss the safety and well-being of participants, progress in intervention, and the fidelity of implementation of the intervention protocol.

#### Implementation Fidelity

Fidelity is rated offline for each type of participant engagement, including with clinicians, peer coaches, and staff coaches. For each type of fidelity rating, at least 30% of files are coded for fidelity, and 20% of these are double coded to ensure interrater reliability. Interrater reliability is computed using Gwet A1C κ. Our predetermined threshold for fidelity is 80% success for program-specific codes across sessions.

#### Plan for Data Analysis

Preregistered data analytic plans are available for review [63] with complementary information available at the clinical trials registration [64]. Plans for power analysis calculations can be found on OSF Registries [63].

Our primary analyses address specific aims and research questions as detailed in Textbox 4.

**Aims and research questions.**

**Aim 1**

Develop and optimize a personalized health algorithm to match neurogenetic condition (NGC) families to digital health support programs using a combination of standard clinical tools and smartphone-based ecological momentary assessment (EMA) data. Primary research questions include the following:

- Does the personalized health algorithm improve the efficacy of support programs in reducing the secondary health effects of COVID-19?
- How effective is each digital health intervention in reducing the secondary health effects of COVID-19 among NGC caregivers?

**Aim 2**

Enhance the scalability and sustainability of digital health interventions by training peer-to-peer coaches to meet the needs of diverse NGC families during the COVID-19 pandemic and beyond. Primary research questions include the following:

- Did peer coaches implement the intervention with fidelity and yield high participant satisfaction?
- Does peer-to-peer coaching improve uptake and efficacy of support programs in reducing the secondary health effects of COVID-19?

Specific analytic strategies are detailed in OSF Registries and are aligned with each research question. Analyses will be led by project biostatisticians who are independent of study design, participant engagement, and algorithm development. To promote rigor and reproducibility, all analytic plans are preregistered on OSF Registries.

#### Interim Data Analysis for Algorithm Optimization

At 2 prespecified intervals, we assess the feasibility, acceptability, and efficacy of the routing algorithm and, broadly, Project WellCAST, in promoting the well-being of caregivers of children with NGCs. Following the CASCADE method for an interdisciplinary “think tank” process, we will use multiple data sources to inform each iteration of the algorithm to optimize and enhance effective triaging for support program implementation [29]. Tables 2-4 detail the metrics of feasibility, acceptability, and efficacy that will be the focus of each think tank, including the desired and undesired outcomes associated with each. Across metrics, we are specifically interested in predicting which individuals will respond suboptimally to the project so that we can proactively minimize these challenges in future versions of the algorithm.

The think tank will be attended by multiple project interest-holders, including NGC caregivers, project staff, clinicians, biostatisticians, and trainees. Before the think tank, biostatisticians will complete preregistered analyses, which will be presented alongside participant feedback and project interest-holder surveys. During the think tank, we will generate a list of hypotheses for how the algorithm can be improved; this list will be narrowed through a systematic process of (1) ensuring each change is supported by more than 2 sources of information (quantitative data, qualitative data, NGC community input, and past literature), (2) evaluating technical feasibility, and (3) ensuring each change is aligned with NGC community priorities. The output of each think tank will be a manuscript submission that justifies specific changes to the algorithm, as well as a preregistration of the resulting optimized algorithm, which will be used to route the subsequent wave of participants to support programs.

**Table 2:** Optimal and suboptimal outcome categories across feasibility metrics^a^.

Feasibility categories		Desired outcome	Undesired outcome	Outcome coding
**Observed feasibility**				
	Nonattempters	Participants consented and started a support program	Participants consented, but dropped out before starting a support program	Behavior
	Noncompleters	Participants started and completed a support program	Participants started a support program, but dropped out before the end of treatment	Behavior
	Low general engagement	Participants completed a support program and completed most of the sessions	Participants completed a support program, but did not complete most of the sessions	Behavior
**Patient-reported feasibility**				
	Low general engagement	Participants completed a support program and were highly engaged throughout	Participants completed a support program, but had low engagement throughout	PATT^b^ items
	Low homework engagement	Participants completed a support program and were highly engaged with homework	Participants completed a support program, but were minimally engaged with homework	Journal log and clinician report

^a^We have retained our original preregistered outcomes but are presenting them via an updated organization.

^b^PATT: Program Acceptability Tool for Telehealth.

**Table 3:** Optimal and suboptimal outcome categories across acceptability metrics

Acceptability categories		Desired outcome	Undesired outcome	Outcome coding
**Patient-reported acceptability**				
	Low acceptability	Participants completed a support program and found the program fit to be highly acceptable based on their needs	Participants completed a support program, but found the program fit to have low acceptability based on their own needs	PATT^a^ total score
**Clinician-reported acceptability**				
	Low acceptability	Participants completed a treatment program, and their clinician rated the treatment program to be a good match based on the participant’s needs	Participants completed a treatment program, and their clinician rated the treatment program to be a poor fit based on the participant’s needs	Clinician rating
**Peer coach-reported acceptability**				
	Low acceptability	Participants were assigned a peer coach, and their peer coach rated WellCAST to be a good fit based on the participant’s needs	Participants were assigned a peer coach, and their peer coach rated WellCAST to be a poor fit based on the participant’s needs	Peer coach rating

^a^PATT: Program Acceptability Tool for Telehealth.

**Table 4:** Optimal and suboptimal outcome categories across efficacy metrics

Efficacy categories		Desired outcome	Undesired outcome	Outcome coding
**Patient-reported efficacy**				
	Nonresponders	Participants initially demonstrated high needs (per the algorithm), completed a support program, and their needs decreased	Participants initially demonstrated high needs (per the algorithm), completed a support program, and their needs persisted	Algorithm data
	Undetected needs	Participants initially demonstrated low needs (per the algorithm), completed a support program, and their needs remained low	Participants initially demonstrated low needs (per the algorithm), completed a support program, and their needs increased	Algorithm data
**Clinician-reported efficacy**				
	Nonresponders	Participants initially demonstrated high needs (per clinician), completed a support program, and their needs decreased	Participants initially demonstrated high needs (per clinician), completed a support program, and their needs persisted	CGI^a^
	Undetected needs	Participants initially demonstrated low needs (per clinician), completed a treatment program, and their needs remained low	Participants initially demonstrated low needs (per clinician), completed a support program, and their needs increased	CGI
**Peer coach-reported efficacy**				
	Nonresponders	Participants initially demonstrated high needs (per coach), completed a support program, and their needs decreased	Participants initially demonstrated high needs (per coach), completed a support program, and their needs persisted	CGI
	Undetected needs	Participants initially demonstrated low needs (per coach), completed a support program, and their needs remained low	Participants initially demonstrated low needs (per coach), completed a support program, and their needs increased	CGI

^a^CGI: Clinical Global Impression Scale.

Additional information about the think tank and CASCADE method procedures is available on OSF Registries and is being published via separate manuscripts that detail the theoretical impetus, process, and outputs of the think tank process.

## Results

**Figure 3:**
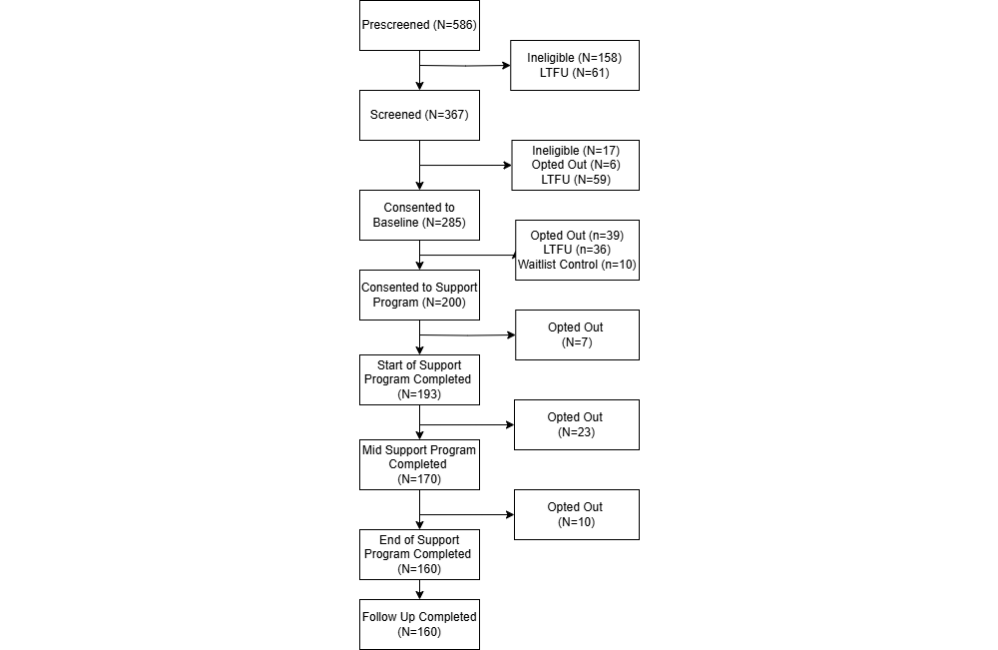
CONSORT (Consolidated Standards of Reporting Trials) flowchart for overall study enrollment as of May 31, 2025. LTFU: loss to follow-up.

The study was approved by the Purdue University IRB on March 3, 2023. The study began its first wave on August 5, 2024, and concluded on January 26, 2024. As of May 31, 2024, 586 participants have completed the virtual prescreening survey. Of those participants, 367 (62.6%) completed screening interviews, and 285 (48.6%) consented to the baseline phase of the study. Figure 3 depicts a CONSORT (Consolidated Standards of Reporting Trials) diagram for additional details regarding initial study enrollment. Data collection is expected to be completed in September 2025, with final study results expected by September 2026; however, the manuscripts will be submitted for publication as data are unmasked across preplanned trial phases.

## Discussion

### Principal Findings

Precision health methods have the potential to improve the feasibility and efficacy of tele–mental health programs for high-need populations, including and beyond NGC caregivers. Project WellCAST tests whether a precision health decision tree algorithm can be developed and used to improve clinical outcomes among NGC caregivers engaging in brief, 12-week, evidence-based support programs. We plan to disseminate trial results through multiple avenues, including publication in peer-reviewed journals, conference presentations, community-based webinars, and other methods as appropriate. It is anticipated that our results will improve understanding of the personal and clinical factors that may predict suboptimal outcomes. If successful, we anticipate Project WellCAST will provide a model for rapidly virtually assessing and triaging populations in need of tele–mental health care, including during future public health emergencies, such as the COVID-19 pandemic.

Several innovations uniquely situate Project WellCAST to contribute to the precision health and psychiatry literatures. First, we use digital health approaches that leverage smartphone-based data to monitor daily fluctuations in symptoms—rather than one-time clinical intake self-report data—providing a more detailed topography of initial patient needs and more sensitive metrics of acute, real-world change over time.

We also leverage community-academic partnerships that provide a scalable, cost-effective model for deploying community-centered interventions while simultaneously training the next generation of mental health providers to deploy digital evidence-based interventions with high-risk populations. To do this, we have aligned multiple digital support programs to a 12-week simultaneous delivery framework, providing a foundation for integrative support programs that can be individualized to families yet delivered with cohesion by trainees.

An additional innovation is that we incorporate extra support elements that we anticipate will boost clinical outcomes for participants who may face further barriers to treatment. For example, we include program options for caregivers experiencing comorbid racial trauma, led by highly qualified experts in the needs of minority families. We leverage community-based partnerships with peer support coaches to deploy evidence-based motivational interviewing protocols to boost program completion and efficacy, while simultaneously maximizing the involvement of patient communities.

These innovations are timely and necessary for NGC caregivers during and after the COVID-19 pandemic; however, this project also has the potential to inform improvements to the long-term landscape for how digital health programs are assigned and deployed far beyond this specific population or public health crisis. Indeed, the high baseline stress and scheduling constraints experienced by NGC caregivers make them an ideal user development group for innovating support program networks that are feasible and acceptable to variety of future users, including those experiencing trauma similar to what was experienced during the pandemic (eg, first responders, teachers, and frontline nurses) or full-time caregivers for other patient groups (eg, patients with dementias). In other words, our model, if successful, can be rapidly adapted and scaled to meet the needs of countless special populations as part of both general standard care and crisis response efforts.

Our study may also inform more cost-effective and sustainable clinical trial practices for several reasons. First, the design of the trial is built on community-academic partnerships, where support programs are deployed by graduate student clinicians with appropriate oversight from licensed supervisors. By engaging student-trainees to deploy support programs, we have minimized the overall cost of deploying programs while simultaneously providing high-quality training opportunities, including necessary clinical hours for licensure eligibility, for the next generation of clinicians. More broadly, our study aims to inform how to minimize mismatches between patient needs and provider offerings, which can reduce unnecessary costs associated with ineffective programming and patient attrition. Appropriately matching patients to support programs can save time, money, and frustrations for both the mental health provider and the patient, supporting sustainability and efficiency.

Project WellCAST was also designed to be intentionally *noninnovative* in 1 important way—the support programs being tested are already evidence-based and widely available to the general population. In other words, it is possible that Project WellCAST’s 2-week baseline data collection procedure and associated precision health algorithm can improve the field’s understanding of how best to route patients to already-available programs in their communities, in line with current directions in precision mental health [14]. For example, our efforts to identify “suboptimal responders” may inform which practical lifestyle factors indicate or counterindicate telehealth-based support programs, which patients may benefit most from additional coaching support, and whether a given patient should be offered mental health support versus parenting coaching. Additional studies on the generalizability of our final decision tree algorithm will be an important interim step toward this goal.

It is important to highlight that we expect Project WellCAST to be a strong match for some participants and a potentially poor match for others. Although telehealth has rapidly expanded in the wake of the COVID-19 pandemic, we and others have continued to caution that telehealth alone will not address the preexisting demographic health disparities that continue to persist across a variety of fields [16]. Our hope is that Project WellCAST can generate the data and results necessary to determine the contexts in which telehealth is most useful [6]. These contributions could be highly clinically relevant for other caregiver groups (eg, other special needs, severe mental illness, and older adults), including those experiencing race-related stress or trauma.

### Conclusions

Project WellCAST tests whether a digital personalized health decision tree algorithm and peer coaching protocol can prospectively enhance telehealth program outcomes among NGC caregivers. This project is relevant to the specific population of NGC caregivers and may also inform how brief digital assessments, precision health tools, and community-academic partnerships can enhance public health response to mental health crises across other high-need populations, during and beyond the COVID-19 pandemic.

## Abbreviations

ACT: acceptance and commitment therapy
CASCADE: community-engaged approach for scientific collaborations and decisions
CONSORT: Consolidated Standards of Reporting Trials
DBT: dialectical and behavioral therapy
EMA: ecological momentary assessment
HIPAA: Health Insurance Portability and Accountability Act
IRB: institutional review board
NGC: neurogenetic condition
OSF: Open Science Framework
REDCap: Research Electronic Data Capture
RUBI: research units in behavioral intervention
SPIRIT: Standard Protocol Items: Recommendations for Interventional Trials
WellCAST: Supporting Well-Being of Caregivers via Telehealth

## Data Availability

Deidentified datasets generated during this study are available from the corresponding author on reasonable request. Data access may be requested for eligible studies and qualified researchers for the purpose of addressing specific scientific objectives. A mutually signed data use agreement (nonnegotiable contract for data use and sharing) must be in place before accessing the requested information.
