# Correction: Optimizing a Personalized Health Approach for Virtually Treating High-Risk Caregivers of Children With Neurogenetic Conditions (Project WellCAST): Protocol for a Randomized Controlled Trial

**DOI:** 10.2196/80023

**Published:** 2025-07-08

**Authors:** Bridgette Kelleher, Kaleb Emerson, Lyndsey N Graham, Veronika Vozka, Anne Wheeler, William Fadel, Daniel Foti, Isha Metzger, Mandy Rispoli, Wendy Machalicek, Laurie McLay, Sean Lane, Wei Siong Neo, Allie Carter, Lisa Brown, Jennifer Brown, Laura Lee McIntyre, Elizabeth Salwitz, Gloria Dietz, Riley Naughton, Katlyn Peek, Nicole Hollins, Emma Woodford

**Affiliations:** 1 Purdue University West Lafayette, IN United States; 2 RTI Research Triangle Park, NC United States; 3 Indiana University Indianapolis, IN United States; 4 Georgia State University Atlanta, GA United States; 5 University of Virginia Charlottesville, VA United States; 6 University of Oregon Eugene, OR United States; 7 Faculty of Health University of Canterbury Ilam, Christchurch New Zealand; 8 University of Missouri Columbia, MO United States; 9 University of Canterbury Ilam, Christchurch New Zealand

In “Optimizing a Personalized Health Approach for Virtually Treating High-Risk Caregivers of Children With Neurogenetic Conditions (Project WellCAST): Protocol for a Randomized Controlled Trial” (JMIR Res Protoc 2025;14:e64360) the authors noted one error.

In Table 1, “Study” column, reference 55 was mistakenly cited as

Kanady and Harvey

55

This has now been corrected to

Durand

55

The correction will appear in the online version of the paper on the JMIR Publications website, together with the publication of this correction notice. Because this was made after submission to PubMed, PubMed Central, and other full-text repositories, the corrected article has also been resubmitted to those repositories.

